# Evaluation of the muscle morphology of the obturator externus and piriformis as the predictors of avascular necrosis of the femoral head in acetabular fractures

**DOI:** 10.1007/s11751-016-0253-7

**Published:** 2016-04-26

**Authors:** Lalit Maini, Santosh Kumar, Sahil Batra, Rajat Gupta, Sumit Arora

**Affiliations:** 1 Department of Orthopaedic Surgery, Maulana Azad Medical College and Associated Lok Nayak Hospital, New Delhi, 110002 India; 2 C/o Mr. Sham Khanna, 2/2, Vijay Nagar, Delhi, 110009 India

**Keywords:** Avascular necrosis, Femoral head, Acetabular fracture, Obturator externus, Piriformis

## Abstract

Avascular necrosis (AVN) of femoral head is a recognised complication of fracture dislocation of the hip joint but is not studied frequently in relation to acetabulum fractures. The aim was to establish the relationship between obturator externus and piriformis muscle morphology in acetabulum fractures and potenital development of AVN of the femoral head. Twenty-five fractures were included in this prospective study and were subjected to radiological assessment and computed tomography of the pelvis. Magnetic resonance imaging (MRI) of the hip was performed to assess the morphology of obturator externus and piriformis, and findings were compared intraoperatively (in 15 cases). Serial radiographs were taken at monthly intervals to assess the development of avascular necrosis. The patients with no evidence of AVN on radiographs at 6 months had additional MRI scans to look for such changes. Three patients developed AVN of femoral head and two had complete tears of piriformis and/or obturator externus muscles on the pre-operative MRI with the findings confirmed intraoperatively (*p* = 0.013). None of the patients without changes of AVN at 6-month follow-up had complete tears of either or both muscles. Of these patients, there was one case each of T-type fracture, isolated posterior wall fracture with hip dislocation, and posterior wall with transverse fracture of the acetabulum. Complete tears of obturator externus and/or piriformis muscles are a strong predictor of future development of AVN of the femoral head.

## Introduction

Acetabular fractures result from high-energy trauma in which soft tissues around the involved hip are severely affected. These cases may, at times, be associated with dislocation of the hip joint and lead to avascular necrosis (AVN) of femoral head potentially. The incidence of AVN has been reported to be 0–11.8 % and attributed as a sequel of the initial dislocation or subsequent to surgery [1–10]. No study has been undertaken, to the authors’ knowledge, to investigate femoral head AVN after acetabular fractures.

**Fig. 1:**
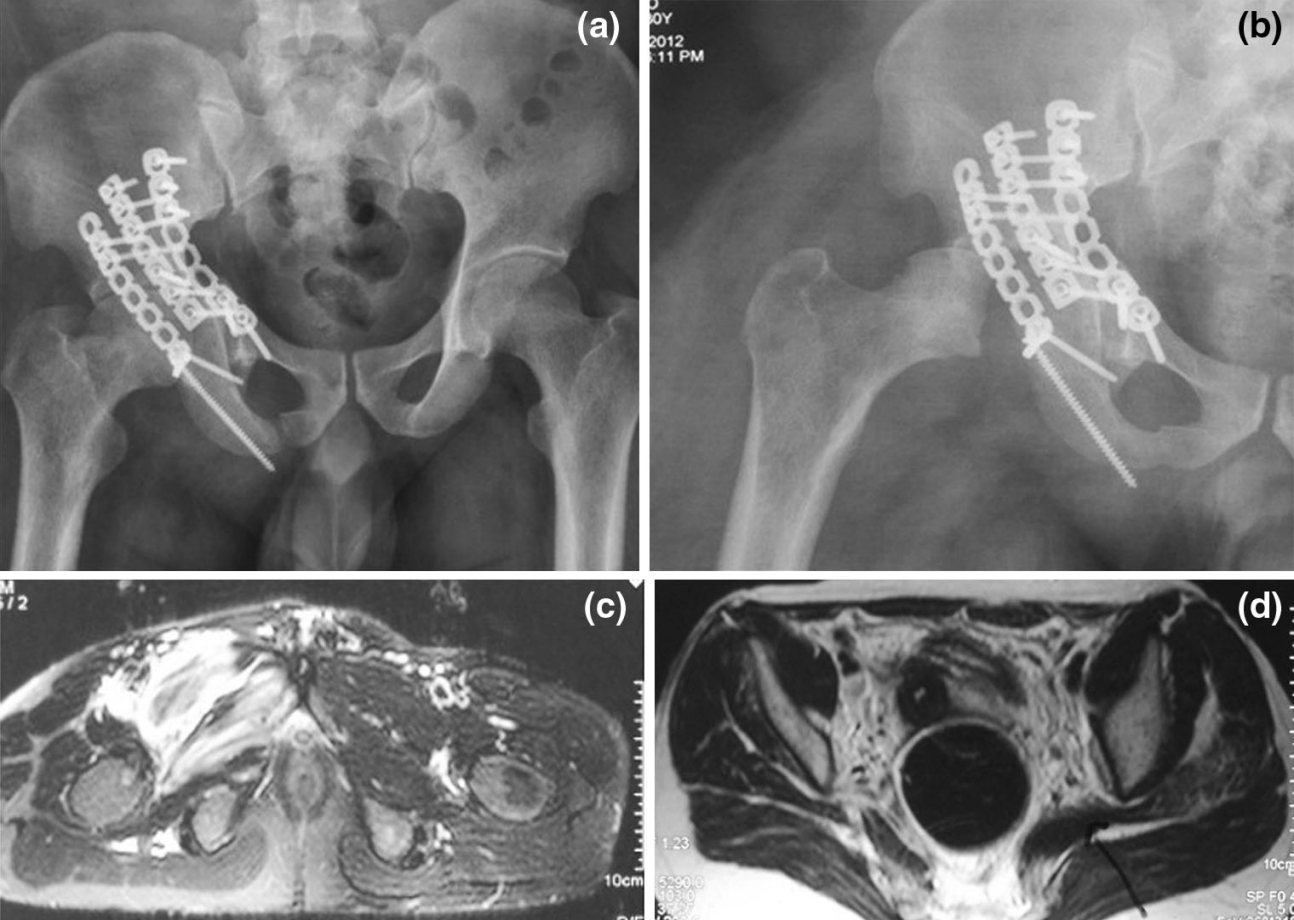
Imaging of case 3 showing T-type acetabular fracture. **a** 2-month post-operative antero-posterior radiograph showing operative fixation of the fracture with reconstruction plates and normal femoral head; **b** 4-month post-operative radiograph showing features suggestive of avascular necrosis of femoral head with resorption; **c** pre-operative MRI of the patient had shown a complete tear of obturator externus; and **d** a partial tear of piriformis

**Fig. 2:**
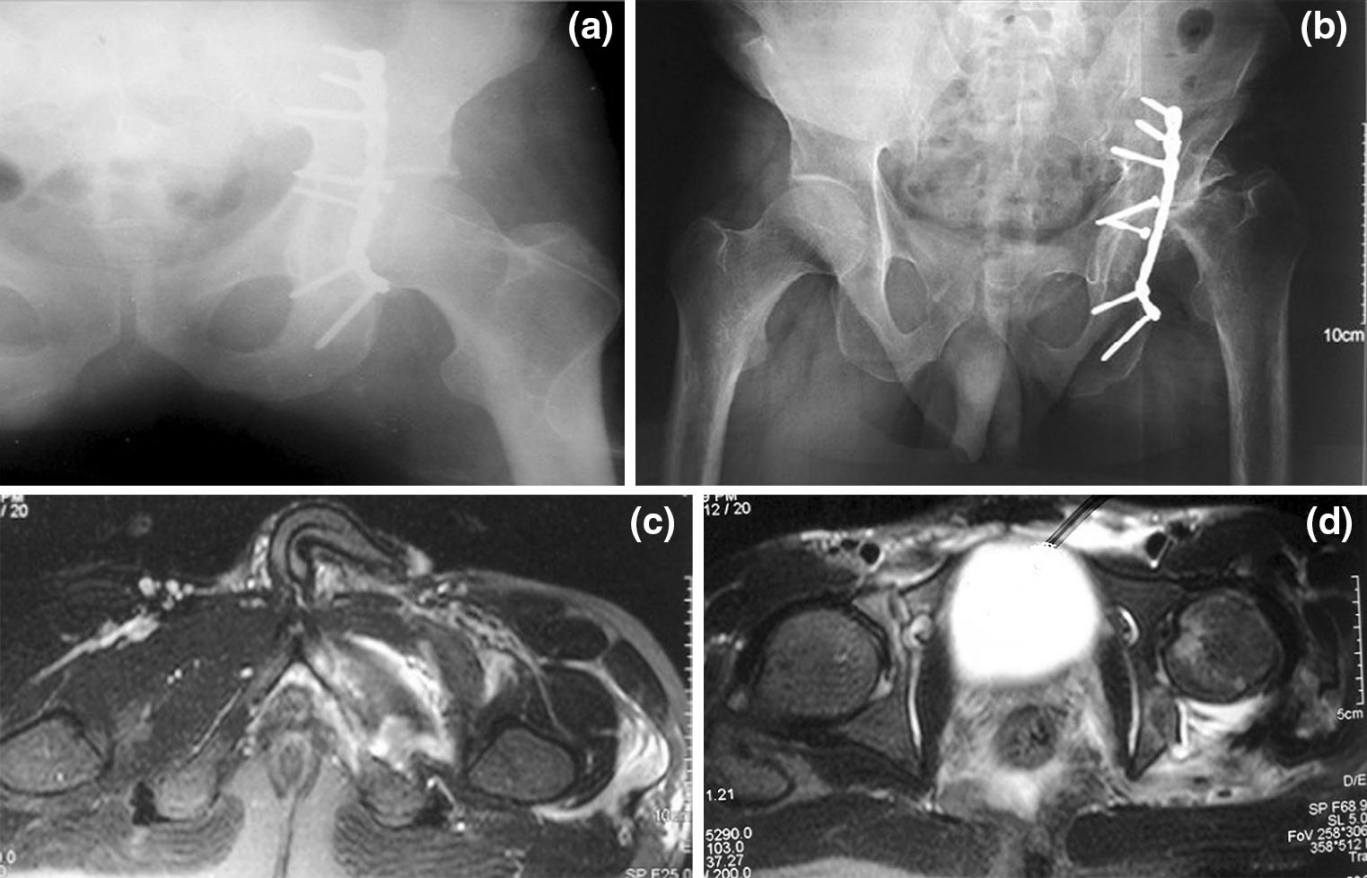
Imaging of case 6 with posterior wall fracture. **a** 1-month post-operative antero-posterior radiograph showing operative fixation of the fracture with buttress reconstruction plate and normal femoral head; **b** 3-month post-operative radiograph showing avascular necrosis of femoral head with resorption; **c** pre-operative MRI of the patient had shown a complete tear of obturator externus; and **d** a complete tear of piriformis

**Fig. 3:**
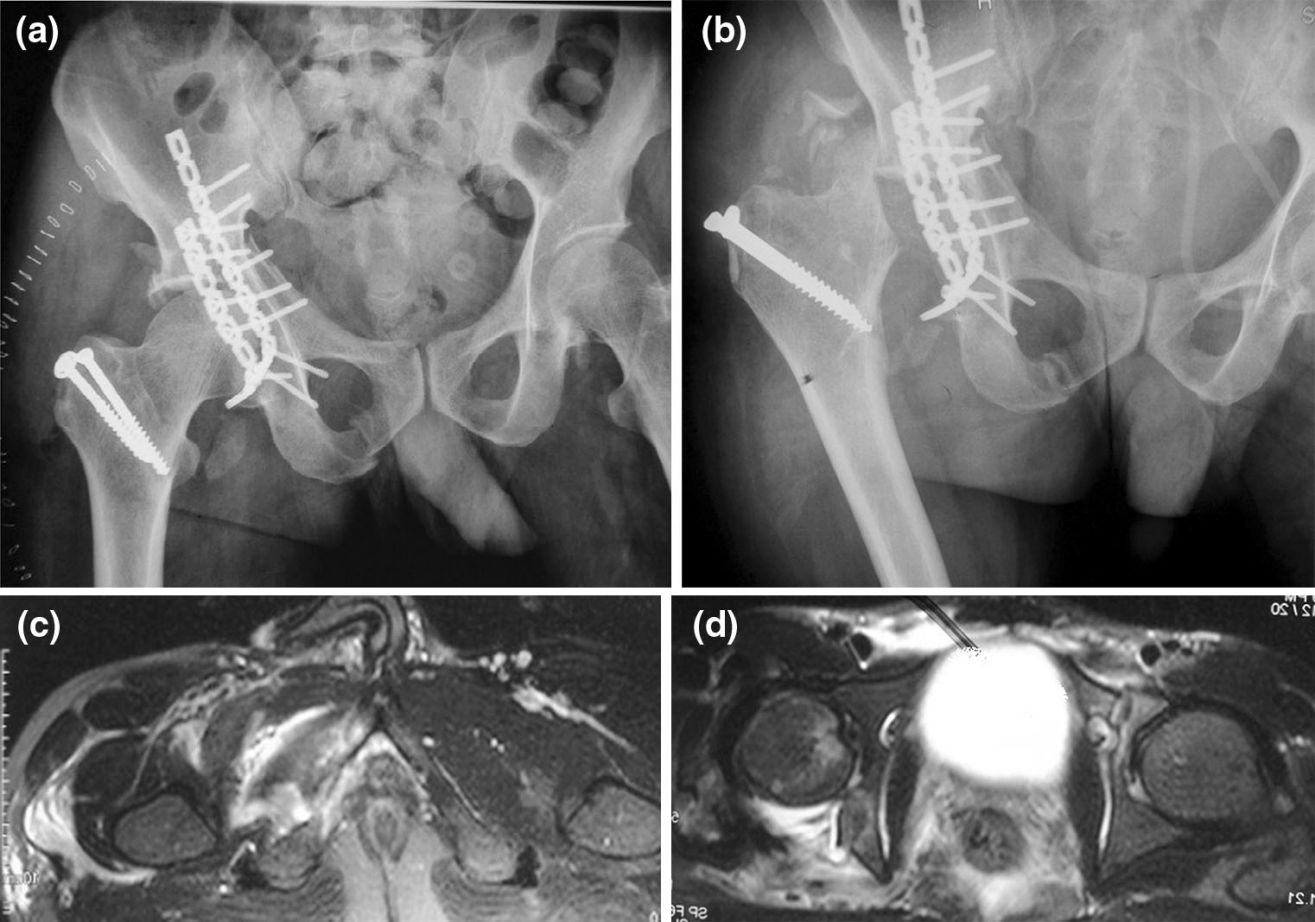
Imaging of case 11 with transverse fracture with posterior wall fracture. **a** Immediate post-operative antero-posterior radiograph showing operative fixation of the fractures with reconstruction plates and normal femoral head; **b** 3-month post-operative radiograph showing evidence of avascular necrosis of femoral head with resorption; **c** pre-operative MRI of the patient had shown a partial tear of obturator externus; and **d** a partial tear of piriformis

The relevant nutrient vessel to the femoral head in adults is the deep branch of the medial femoral circumflex artery (MFCA) [11]. It may be injured during the posterior approach to the hip and acetabulum. There are two central and five peripheral anastomoses of the MFCA [11]. All of the peripheral anastomoses have been observed to be extracapsular with the largest and the most consistent being a branch of the inferior gluteal artery which runs along the inferior border of piriformis. Gautier et al. [11] stated the piriformis branch might play a role in vascularisation of the femoral head after injury of the deep branch of the MFCA. They observed also the obturator externus muscle protects the MFCA when the hip is dislocated. Theoretically, an intact obturator externus tendon would imply a preserved blood supply to the femoral head if no additional intracapsular lesion of the MFCA is present.

We sought to evaluate the possible association between the integrity of obturator externus and piriformis muscle and femoral head vascularity in acetabular fractures prospectively. We investigated the possible association of acetabular fracture pattern on development of AVN of the femoral head.

## Patients and methods

The study was undertaken in a government-based tertiary care teaching hospital between July 2011 and April 2013. All consecutive patients presenting with acetabular fractures were evaluated by two authors (LM and SK) for possible inclusion in the study by the following criteria: (1) closed pelvic fractures; (2) age group 18–60 years; (3) injury less than 2 weeks old; (4) adequate pelvis radiographs and Judet views. All patients with pre-existing hip disease, ipsilateral fracture of the head and neck of the femur, inter-trochanteric fracture, or with any contraindication for an MRI examination were excluded. Those with fractures associated with life-threatening complications were excluded also. There were 25 patients who met the requirements. Written informed consent was obtained from each patient and approval from the Institutional Review Board granted.

These patients had non-contrast computed tomography (NCCT, with 3-dimensional reconstructions) and plain radiographs to evaluate the anatomy and type of fracture. Magnetic resonance imaging (MRI) of the pelvis and both hips were performed to assess the integrity of obturator externus and piriformis muscles. The scans were obtained with an advanced high-field 1.5-T scanner (Sonata Magnetom, Siemens) using the standard protocol. The muscle injury was graded as proposed by Rybak et al. [12]. The system included grade 1 (oedema only no discontinuity of fibres—contusion); grade 2 (partial tear); and grade 3 (complete tear) pattern of muscle injury.

The mode of treatment (non-operative or operative) was decided on an individual basis after evaluating the general condition, fracture displacement, hip joint stability, and congruity. All cases were operated by an experienced pelvi-acetabular trauma surgeon (LM). The appropriate surgical approaches were chosen based on the fracture pattern and surgeon’s experience. The clinical morphology of obturator externus and piriformis could not be evaluated for the patients that were managed non-operatively or those operated with an anterior approach alone. Fracture fixation was done using a variety of 3.5-mm reconstruction plates, 6.5-mm cancellous lag screws, 4.0-mm cancellous lag screws, and 3.5-mm cortical screws (lengths up to 120 mm) and 6.5-mm fully threaded cancellous screws depending on fracture pattern. All the implants were made of titanium metal so that an MRI could be done 6 months post-operatively if required.

The reduction of the fracture fragments was evaluated post-operatively with similar antero-posterior and Judet view radiographs. The immediate post-operative reduction was evaluated and graded according to radiological grading as described by Matta and Merritt [13]. Patients were kept non-weight bearing for a period of 6–12 weeks depending on the stability of fixation. Full weight bearing was allowed after 12–20 weeks after surgery. Serial radiographs were taken at monthly interval to assess the fracture healing and detect the development of avascular necrosis of femoral head. All patients who had normal femoral head appearances on radiographs at the end of 6 months had further MRI evaluation for development of avascular necrosis of femoral head. The functional status was assessed at 6 months according to Merle d’ Aubigne scoring system [14].

The analysis of data was performed using Statistical Package for Social Science (SPSS Inc. version 17.0 for windows) Chicago, Illinois. Fisher’s exact test was used to examine the significance of association (contingency) between two groups. It was referenced for a two-tailed *p* value, and a 95 % confidence interval was constructed around sensitivity proportions using normal approximation method. A two-tailed *p* value of <0.05 was assumed to attain sufficient statistical significance.

## Results

Twenty-five patients were evaluated in this present study and included 22 men and 3 women. The mean age of patients was 31.8 years (range 18–55 years). The most common fracture pattern was a T-type (eight patients, Table 1). Isolated posterior column and anterior wall fractures were not detected in any of the patients. Fifteen patients underwent surgery and 10 patients were treated non-operatively. The Kocher–Langenbeck approach was used in eight cases, the ilio-inguinal approach in five cases, and a combined approach for two cases. Intraoperatively, the morphology of piriformis and obturator externus was examined in 10 cases that were operated with Kocher–Langenbeck approach and the findings compared with the pre-operative MRI.

**Table 1:** Detailed outline of all the 25 patients in our series

S no	# Type	Treatment	Delay in surgery (days)	Surgical approach	MRI findings	Intraoperative findings
1	Transverse	Operative	3	Posterior	Contusion/partial tear both muscles	Same
2	Transverse	Non-operative	–	–	Both intact	–
3	T-type	Operative	12	Ant + post	Complete tear either/both	Same
4	Ant + post hemi transverse	Operative	4	Anterior	Both intact	–
5	T-type	Operative	3	Anterior	Contusion/partial tear both muscles	–
6	Post wall	Operative	4	Posterior	Complete tear either/both	Same
7	Post wall	Non-operative	–	–	Both intact	–
8	Transverse	Operative	7	Anterior	Contusion/partial tear obturator externus	–
9	Posterior wall	Operative	5	Posterior	Both intact	Same
10	T-type	Operative	5	Posterior	Contusion/partial tear obturator externus	Same
11	Post wall + transverse	Operative	3	Posterior	Contusion/partial tear both muscles	Same
12	Bicolumnar	Operative	3	Posterior	Contusion/partial tear piriformis	Contusion/partial tear both muscles
13	Transverse	Operative	8	Posterior	Contusion/partial tear both muscles	Same
14	Transverse	Non-operative	–	–	Both intact	–
15	Ant column	Non-operative	–	–	Both intact	–
16	T-type	Operative	3	Posterior	Contusion/partial tear both muscles	Same
17	Ant column	Operative	8	Anterior	Contusion/partial tear both muscles	–
18	Bicolumnar	Operative	4	Anterior	Contusion/partial tear both muscles	–
19	T-type	Operative	7	Ant + post	Contusion/partial tear both muscles	Same
20	Ant column	Non-operative	–	–	Both intact	–
21	T-type	Non-operative	–	–	Both intact	–
22	Transverse	Non-operative	–	–	Both intact	–
23	T-type	Non-operative	–	–	Both intact	–
24	Post column + post wall	Non-operative	–	–	Both intact	–
25	T-type	Non-operative	–	–	Contusion/partial tear both muscles	–

Patient number 3, 6, and 11 developed AVN of the femoral head in follow-up

The MRI revealed complete tears of either or both piriformis and obturator externus in two patients, a partial tear of both muscles in nine patients, an isolated partial tear of obturator externus in two patients, and an isolated partial tear of piriformis in one patient. The remaining patients had intact or partially contused muscles (Table 1). Avascular necrosis was seen in both patients with complete tears of either or both piriformis and obturator externus. However, only one patient developed avascular necrosis out of nine patients who had partial tear of both the muscles (Table 2). The remaining patients who had normal or partially contused muscles or partial tears of one of the muscles did not develop femoral head AVN. Of the remaining 22 patients who did not develop avascular necrosis of the femoral head, eight patients had contusion or partial tears of both piriformis and obturator externus muscles on MRI and half of these were confirmed intraoperatively. The other eight patients had both the muscles declared intact on MRI, but intraoperative confirmation of these findings was not possible as they were managed non-operatively. The pre-operative grading of obturator externus and piriformis tear as a predictor of future AVN of the femoral head was statistically significant (*p* = 0.013). Only one patient was found to have different muscle morphology intraoperatively in comparison with the pre-operative MRI.

**Table 2:** Table showing detailed outlines of the patients that developed AVN of the femoral head in follow-up

No.	Age (Y)	Sex	Type of fracture	MRI status of obturator ext. and piriformis	Management	Time since injury to surgery (days)	Associated dislocation	Approach	Intraoperative muscle status
3	32	M	T-type (Fig. 1a–d)	Complete tear of obturator externus, partial tear of pyriformis	Operative	12	Posterior	Combined	Same as MRI findings
6	42	F	Post. Wall (Fig. 2a–d)	Complete tear of both the muscles	Operative	4	Nil	Posterior	Same as MRI findings
11	42	M	Post. wall + Transverse (Fig. 3a–d)	Partial tear of both the muscles	Operative	3	Central	Posterior	Same as MRI findings

An associated hip dislocation was found in seven patients of which two developed AVN of the femoral head. With the numbers available, hip dislocation as a predictor of future development of femoral head AVN was not statistically significant (*p* = 0.18). Of the three patients with posterior wall fractures, one developed AVN of the femoral head and one patient (out of eight) with T-type fractures developed this complication. The only patient in the study with a transverse with posterior wall fracture also developed AVN of the femoral head. Ten patients were managed non-operatively and none of them had evidence of AVN of the femoral head in follow-up. However, the mode of treatment as a predictor for future development of AVN was not statistically significant (*p* = 0.25).

## Discussion

Avascular necrosis of the femoral head after acetabular fractures is a devastating outcome. There is little information on prognosis of this complication in this patient group. As was suggested from earlier studies [11], we agree that both piriformis and obturator externus muscles protect the MCFA which is the major blood supply to the head of the femur. In this study, both patients who had complete tears in either or both obturator externus and piriformis muscles developed AVN of the femoral head (*p* = 0.013). Thus, an injury to obturator externus and piriformis muscles may be indirect pointer for damage to the MCFA and, potentially, subsequent development of AVN of the femoral head.

Tannest et al. [15] performed MRI scans in the acute phase of posterior hip dislocation and suggested that an intact obturator externus tendon preserves the deep branch of MFCA to the femoral head. In accordance with this suggestion, we propose that early MRI evaluation may help detect injury to obturator externus or piriformis muscles and potential development of AVN of the femoral head at an early date and avoid the erroneous link to an iatrogenic complication of the surgical approach to the acetabulum [16]. Of the 10 patients treated conservatively, none developed avascular necrosis. This is in keeping with the earlier studies but, in this study, this mode of treatment as a predictor of avascular treatment was not statistically significant. In contrast, in the posterior approach to the hip and acetabulum where tenomyotomy of the external rotator muscles is done, this interrupts the anastomosis between the inferior gluteal artery and the deep branch of the MFCA and subjects the deep branch itself to risk.

The finding in all the cases that developed avascular necrosis of femoral head was of significant head resorption; this is not seen in a traumatic osteonecrosis of femoral head. Other causes which could lead to head resorption are occult fracture of femoral head or infection. However, in this study, an occult head fracture was ruled out in all cases since pre-operative CT and MRI evaluations were undertaken. Infection was ruled out by hip aspiration and culture which was repeated when these patients had total hip replacements.

Previous studies [1–10, 17–19] on acetabular fractures reported the percentage of cases with avascular necrosis (Table 3). The majority did not comment as to what may have led to avascular necrosis and none noted the extensive head resorption. Further studies with larger sample sizes are needed to evaluate all possible predictors of AVN of the femoral head after acetabular fractures. We suggest pre-operative MRI scans of the pelvis may be considered for high-energy trauma cases that require operative stabilisation. Such an exercise may be helpful in pre-operative prognostication for the development of avascular necrosis of the femoral head.

**Table 3:** Various reported series on acetabular fractures with possible remarks on the development of AVN of the femoral head following this injury

S. no.	Author	Year	No. of cases	% with AVN	Hypothesis given	Remarks
1	Matta [18]	1988	121	0	No	–
2	Heeg [8]	1990	54	11.2	No	–
3	Mayo [3]	1994	163	0.6	No	–
4	Matta [2]	1996	259	3	No	–
5	Siebenrock [6]	2002	12	0	Yes	Obturator externus acts as a protector for deep branch of MCFA
6	Giannoudis [4]	2005	2010	5.6	No	–
7	Panagiotis [7]	2007	75	8	No	–
8	Hadjicostas [5]	2008	31	6.4	No	–
9	Tannast [1]	2010	60	0	Yes	Injury to MCFA
10	Naranje [9]	2010	18	5.5	No	–
11	Briffia [10]	2011	161	11.8	No	–
12	Uchida K [16]	2012	91	0.022	No	–
13	Mitsionis [17]	2012	19	0.053	Yes	Greater chance of AVN in isolated posterior dislocation of the hip than dislocation with fracture More chances of AVN if head is reduced late

## Conclusion

We conclude that damage to obturator externus and piriformis is a possible predictor for consequent development of AVN of the femoral head which can be judged on pre-operative MRI scans. The type of acetabular fracture and associated dislocation of the hip, if present, may have a bearing on this complication but was not established in this sample studied.
